# Building *invitro* 3D human multicellular models of high-grade serous ovarian cancer

**DOI:** 10.1016/j.xpro.2021.101086

**Published:** 2022-01-11

**Authors:** Beatrice Malacrida, Oliver M.T. Pearce, Frances R. Balkwill

**Affiliations:** 1 Centre for Tumour Microenvironment, Barts Cancer Institute, Queen Mary University of London, Charterhouse Square, London EC1M 6BQ, UK

**Keywords:** Cancer, Cell Biology, Cell culture, Cell isolation

## Abstract

Three-dimensional (3D), multicellular *invitro* models provide a useful platform for studying human cancer biology, particularly through deconvolution of the tumor microenvironment, or where animal models do not recapitulate the human condition. Here, we detail a protocol for building human multicellular models made of patient-derived primary cells and malignant cell lines, which recapitulate features of the tumor microenvironment. This protocol is optimized for building 3D models of high-grade serous ovarian cancer omental metastasis but can be adapted for modeling other cancers.

For complete details on the use and execution of this profile, please refer to Delaine-Smith et al. (2021) and Malacrida et al. (2021).

## Highlights

• Isolation of fibroblasts and mesothelial and adipocyte cells from omental tissue
• Steps for casting adipocyte cells in collagen-based gels
• Building tri-culture (three cell types) and tetra-culture (four cell types) *in vitro* models
• *In vitro* models with patient-derived primary cells and HGSOC malignant cells

## Graphical abstract

**Figure:**
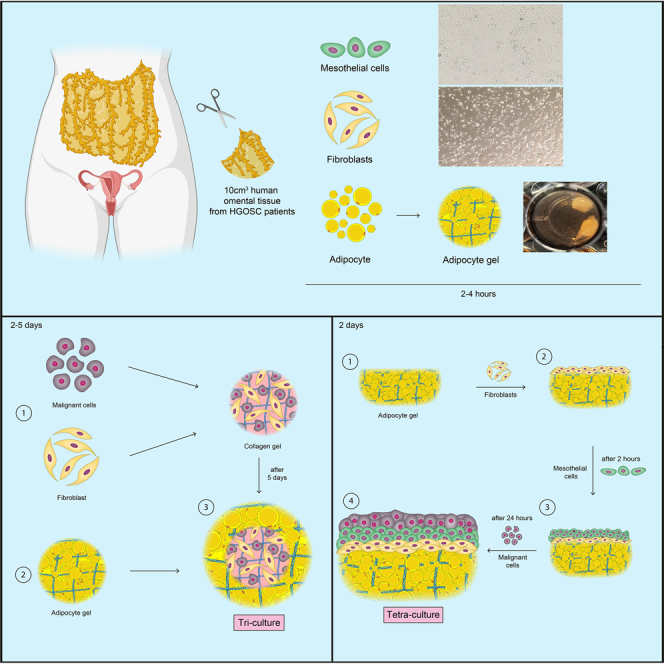

## Before you begin

The protocol below describes the isolation and construction of human in-vitro multicellular models using fresh omental tissue and high-grade serous ovarian cancer, HGSOC, malignant cell lines. However, we think this protocol could be used to build similar tissues for other cancers especially those that develop omental metastases such as colorectal cancers.

The omental tissue used in this protocol comes from a surgical or diagnostic biopsy that is surplus to requirements. Approval by a UK national review board and written consent from patients were obtained (HTA license number 12199. REC no: 10/H0304/14).

### Omental tissue processing

**Timing: 2–4 h**

Omental tissues were collected from patients undergoing surgery and placed in 0.9% saline solution in a sealed container. The container was then placed in a sealed box containing ice and transported immediately to the lab. The volume of saline solution used depends on the size of tissue. The tissue collected needs to be covered with saline solution.

This protocol is optimized for processing 10 mL of tissue, so all the volumes and concentrations given are based on this quantity.

1. Mesothelial cell isolation
2. Stromal vascular fraction isolation (including fibroblasts)
3. Adipocyte isolation
  a. Adipocyte gel casting

***Note:*** To obtain a higher cell yield, it is advisable to process the tissue as soon as possible and no longer than 24 h from de-vascularization during the surgery.

### Tri-culture or tetra-culture preparation

**Timing: 2–7 days**

4. Tri-culture set up
  a. Collagen gel casting
  b. Collagen and adipocyte gel embedding

Or

5. Tetra-culture set up
  a. Fibroblast and mesothelial cells addition to the adipocyte gel
  b. HGSOC cell line addition to the model

***Note:*** Adipocyte gels, tri- and tetra-culture models can be handled using a small spoon or spatula.

***Note:*** Add fibroblasts, mesothelial cells, and tumor cells 24–48 h after casting the adipocyte gel to allow the adipocyte to set within the collagen gel.

***Note:*** A good indicator of healthy adipocyte cultures is the production of small oil droplets released into the medium. Healthy adipocyte gels will look round and yellow/white in color.

## Key resources table

**Table:** 

REAGENT or RESOURCE	SOURCE	IDENTIFIER
**Chemicals, peptides, and recombinant proteins**		

Trypsin-EDTA solution 10×	Sigma-Aldrich	Cat#T4174
DMEM/F12 with Glutamax	Thermo Fisher Scientific	Cat#31331093
Penicillin/Streptomycin	Fisher Scientific	Cat#11548876
FBS	Fisher Scientific	Cat#10500-064
Collagenase type I powder	Thermo Fisher Scientific	Cat#17100017
L-Ascorbic acid 2-phosphate sesquimagnesium salt hydrate	Sigma-Aldrich	Cat#A8960
Collagen I from rat tail	Thermo Fisher Scientific	Cat#A1048301
DMEM low glucose 10×	Sigma-Aldrich	Cat#D2429
Medium-199	Thermo Fisher Scientific	Cat#22350029
DPBS, no calcium, no magnesium	Thermo Fisher Scientific	Cat#14190144
Insulin-Transferrin-Selenium-Sodium Pyruvate (ITS-A) (100×)	Thermo Fisher Scientific	Cat#51300044

**Biological samples**		

Human Omental fresh tissue	Barts Health NHS Trust	N/A

**Experimental models: Cell lines**		

Human High Grade Serous Ovarian Cancer cells	isolated in our lab	(Tamura et al., 2020)

**Other**		

Pierce™ Tissue Strainers, 250 μm, 2.5 mL	Thermo Fisher Scientific	Cat#87791
Disposable scalpels	Swann Morton	Cat#0501
Innova 40 shaker incubator	Eppendorf	N/A

**Software and algorithms**		

BioRender	BioRender	Some graphical abstract components were created with https://biorender.com/

## Materials and equipment

**Table:** Tissue medium

Reagent	Final concentration	Amount
DMEM:F12 with GlutaMAX	n/a	95 mL
FBS	5%	5 mL

***Alternatives:*** Normal DMEM:F12 can also be used without any glutamine addition.

**Table:** Culture medium

Reagent	Final concentration	Amount
DMEM:F12 with GlutaMAX	n/a	445 mL
FBS	10%	50 mL
Penicillin/Streptomycin	1%	5 mL

**Table:** Adipocyte gel mixture

Reagent	Final concentration	Amount (example)
Rat tail collagen I (3 mg/mL)	1 mg/mL	1 mL
DMEM low glucose 10×	1×	300 μL
dH_2_O	n/a	650 μL
NaOH 1M	5 μL every 100 μL of collagen	50 μL
Adipocyte	n/a	1 mL
**Total**	**n/a**	**3 mL**

***Note:*** The total volume of the solution is based on the volume of adipocytes isolated during the protocol.

***Note:*** All the steps involving the adipocyte gel preparation need to take place on ice.

**CRITICAL:** For the adipocyte gel mixture, add the reagents in the order shown in the table (top to bottom). NaOH will start the polymerization reaction of the collagen. The speed of polymerization can be decreased by keeping the gel mix on ice. The amount of NaOH used for every collagen mix needs to be titrated. We recommend NaOH be added drop wise until the solution turns pink. Too much NaOH will lower the pH and reduce cell viability. Too little NaOH may prevent proper setting of the collagen gel.

***Alternatives:*** MEM 10× with no glutamine can be used in place of DMEM low glucose 10×.

**Table:** Adipocyte gel medium

Reagent	Final concentration	Amount
M199	n/a	445 mL
FBS	10%	50 mL
Penicillin/Streptomycin	1%	5 mL

**Table:** Collagen gel mixture

Reagent	Final concentration	Amount
Rat tail collagen I (3 mg/mL)	1 mg/mL	33 μL
DMEM low glucose 10×	1×	5 μL
NaOH 1M	5 μL every 100 μL of collagen	1.7/2 μL
Cells + Medium	n/a	60 μL
**Total**	**n/a**	**100 μL**

**Table:** Collagen “adhesive” solution

Reagent	Final concentration	Amount
Rat tail collagen I (3 mg/mL)	1 mg/mL	100 μL
DMEM low glucose 10×	1×	30 μL
dH_2_O	n/a	165 μL
NaOH 1M	5 μL every 100 μL of collagen	5 μL
**Total**	**n/a**	**300 μL**

## Step-by-step method details

### Omental tissue processing

**Timing: 2–4 h**

This first step of the protocol will allow the isolation of mesothelial cells, fibroblasts and adipocytes from fresh omental tissue, as also described by other authors (Carswell et al., 2012; Toda et al., 2009; Sonoda et al., 2008). Tissue and culture media should be warmed in a 37°C water bath until use.

***Note:*** Fresh tissue should be covered in 0.9% saline solution and left at 4°C until processing. After the surgical resection, all the steps of this protocol should be performed under a laminar flow hood.

1. Isolation of mesothelial cells
  ***Note:*** To obtain the best yield of mesothelial cells, it is recommended to process the tissue as soon as possible and no longer than 24 h from resection.
  a. Add 10 cm^3^ (10 mL measured in a 50 mL falcon tube) of tissue to a 20 mL 1:1 solution of 10× trypsin and 1× DPBS in a 50 mL falcon tube.
  b. Place the tube in a 37°C water bath for 20 min. Gently invert the tube twice after 10 min and at the end of the incubation period.
  c. Neutralize the solution by adding 20 mL of warm tissue medium.
  d. Remove the tissue and place it into a 10 cm petri dish.
  e. Spin down the solution in the falcon tube at 200 g for 5 min at room temperature. The pellet consists of the mesothelial cells, which can be resuspended in culture medium and plated in a flask or petri dish.
    ***Note:*** The mesothelial cell pellet will appear bloody and cloudy. Do not aspirate off all the supernatant, leave 2–3 mL of supernatant in the falcon tube. Resuspend the pellet with culture medium and plate everything in a T75 flask or 10 cm petri dish. Leave the mesothelial cells in this medium for at least 2–3 days to allow the cells to attach to the plate. After 2–3 days, remove the spent medium, wash with 1× DPBS to remove any debris and add new fresh medium.
    ***Note:*** To check the quality and purity of the isolation, leave the cells growing for at least a week. Mesothelial cells have a cobblestone morphology and are positive for cell surface marker calretinin (Figure 1A).
    **Figure 1:**
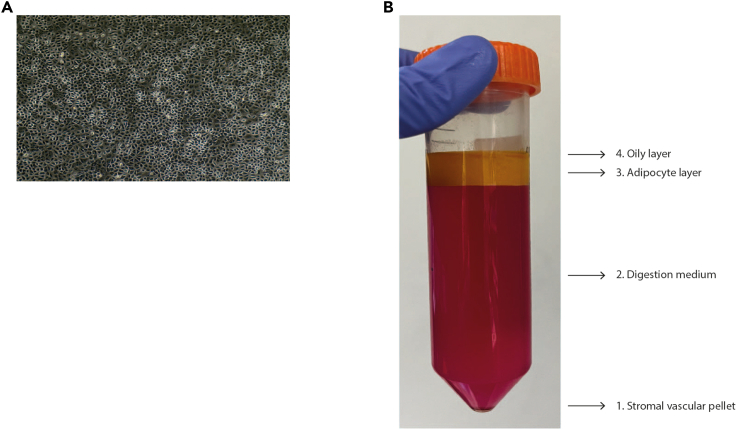
Stages of cell isolation from omental tissue (A) Mesothelial cell line image taken at 10×. (B) Different phases after filtration of the digested tissue.
  f. Use two scalpels (or scissors) to mince the tissue from step 1d in small pieces (no bigger than 1 mm^3^).
  g. Add the minced tissue to a 20 mL 1 mg/mL collagenase solution prepared in warm tissue medium to digest the tissue. Use a 50 mL falcon tube to obtain an appropriate digestion.
  h. Incubate the minced tissue and collagenase solution in a shaker at 37°C, 65 g for 30–60 min, until the solution is cloudy and the tissue digested.
    ***Note:*** The incubation time will depend on the tissue density. If the tissue is very fatty and soft, incubate for only 30 min. In case of a very fibrotic and stiff tissue, incubate up to 60 min to allow proper tissue digestion.

2. Isolation of the stromal vascular fraction
  a. After 1 h incubation, mix gently the digested tissue using a plastic Pasteur pipette with the end cut off until complete dissociation of all the tissue clumps.
    ***Note:*** Using the Pasteur pipette, you can try to break down some clumps of tissue, which will form during the digestion.
  b. Filter the digested tissue using 250 μm strainers that fit into a 15 mL falcon tube. Using the plunger of a 1 mL syringe, gently press the tissue on the filter to help the filtration process.
  c. The filtered solution will present four phases which starting from the bottom of the falcon tube are: 1) the pellet that contains the stromal vascular components; 2) pink/red spent digestion medium; 3) a yellow/white layer of adipocytes and 4) a top oily layer containing released lipids and adipocyte debris (Figure 1B).
  d. Once you have 2–3 mL of the filtered solution in the 15 mL falcon tube, gently pipette the filtered solution (both fractions) into a new 50 mL falcon tube containing 20 mL of warm tissue medium to wash inactivate any residual of collagenase. You can then continue to filter the digested tissue using the same 250 μm strainer.
    ***Note:*** It is very important not to leave the filtered solution for too long in the 15 mL falcon tube, as the remaining collagenase can affect the viability of the adipocyte fraction. Over-digested tissue will appear slimy/almost liquid.
  e. After filtering all the digested tissue, spin down the 50 mL falcon tube containing the four fractions and the fresh tissue medium at 200 g for 3 min at room temperature. Dispose appropriately all the tissue left after the digestion.
  f. After the centrifugation, do not aspirate off the supernatant, but move carefully (using a plastic Pasteur pipette) the fraction containing the adipocytes (second fraction from the top) to a new 50 mL falcon tube with 20 mL of warm fresh tissue medium, without disturbing the pellet present in the below pink/red fraction.
  g. Once the adipocyte fraction has been moved to the new 50 mL falcon tube, aspirate the remaining supernatant without disturbing the pellet. This pellet contains the stromal vascular fraction, which consists mainly of fibroblasts.
  h. Resuspend the pellet in fresh culture medium and plate the cells in a flask/petri dish.
    ***Note:*** The stromal vascular fraction solution will appear bloody and cloudy. Once the cells are plated in a 10 cm petri dish/T75 flask, incubate at 37°C, 5% CO_2_ the cells in this medium for at least 2–3 days to allow the cells to attach to the plate. After 2–3 days, remove the spent medium, wash with 1× DPBS to remove any debris and add new fresh medium.
    ***Note:*** To check the quality and purity of the isolation, leave the cells growing for at least a couple of days. You can test their phenotype by staining them with any of the common markers used for fibroblast, such as α-smooth muscle actin (α-SMA), FAP (fibroblast activation protein alpha) and others.

3. Adipocyte isolation
  a. Spin down the falcon tube containing the adipocyte fraction from step 2f at 200 g for 2 min at room temperature.
  b. To separate properly the adipocyte fraction from the residual medium/red fraction, use a plastic Pasteur pipette and try to collect only the adipocyte fraction which is the second fraction from the top, avoiding the oily top fraction and the spent bottom medium as much as possible. Place the adipocyte fraction in a new 15 mL falcon tube.
    ***Note:*** It is very important to minimize the uptake of excess medium. It is also very critical to avoid the oily fraction, which will be present just above the adipocyte (yellow) fraction. Failing to do so will affect the proper polymerization of the gel.
  c. Repeat step 3b until the adipocyte fraction is the only fraction present within the tube.
    ***Note:*** To easily separate the remaining medium from the adipocyte fraction, leave the tube in a rack for a couple of minutes, until the phases will separate.
  d. Measure the volume of the adipocyte fraction and prepare the adipocyte gel mixture as indicated in the Methods.
    ***Note:*** While preparing the adipocyte gel mixture, all the components, excluding the adipocyte fraction, will need to stay in ice, until adipocyte addition.
  e. Once the adipocyte gel mixture is prepared, pipette gently 100 μL of solution in 96-well plate. Invert the tube every 30 s (or roughly every 4 gels plated) to mix the solution. Incubate immediately at 37°C, 5% CO_2_ to facilitate gel polymerization.
    ***Note:*** Step 3e has to be done quickly with care. It is important that gels set quickly to avoid different components separating within the mixture.
  f. Incubate for at least 30 min. Once solidified, the gels will look yellow/white. Using a very small spoon or spatula, carefully transfer each gel to a 24-well Corning plate containing 1 mL of adipocyte gel medium. Gels will float in the medium. Medium change is required every second day. The adipocyte gels are viable and usable for further experiments up to 24–30 days.

### Tri- or tetra-culture preparation

**Timing: 2–5 days**

This part of the protocol will allow the set-up of two different 3D human multicellular models (tri- and tetra-culture i.e., three and four cell type cultures). All the steps should be performed in a laminar flow hood.

4. Tri-culture set up
  The tri-culture model is made of three different cell lines: HGSOC malignant cell lines, omental fibroblasts (isolated following point 2 of this protocol) and adipocyte gels (prepared in point 3 of this protocol). This model consists of a collagen gel made up of malignant and fibroblast cells that is then embedded within an adipocyte gel.
  a. To set up the collagen gel, follow the recipe of the collagen mixture listed in the methods. The ratio between fibroblast and cancer cells used is 1:1 (for our model we used 1 × 10^5^ to 2.5 × 10^5^ cells)
    ***Note:*** The cell ratio within the collagen gel might vary according to the type of cancer studied, the aggressiveness of the malignant cells and their duplication time.
    i. Once the collagen gel mixture is ready, pipette 100 μL of the solution in a 96-well plate. Incubate the collagen gels for at least 1 h in a 37°C, 5% CO_2_ incubator.
      ***Note:*** Once solidified, the collagen gels will appear white/transparent. The collagen gels are fragile, take extra care when moving them.
    ii. After the incubation, use a small spatula or spoon to detach the gels from the 96-well plate and to move them in a 24-well plate with 1 mL of culture medium. Medium needs to be changed every second day.
      ***Optional:*** To increase extracellular matrix (ECM) production by both fibroblasts and malignant cells, it is possible to add a 50 μg/mL ascorbic acid solution in the medium every second day.
    iii. Collagen gels can be incorporated in the adipocyte gels after 5–7 days of incubation.
  b. To incorporate the collagen gel within the adipocyte gel, gently move an adipocyte gel from the culture plate to the bottom of a 24-well plate (no medium required).
    i. Quickly take a collagen gel from the culture plate and place it on top of the adipocyte gel.
    ii. Gently press the collagen gel within the adipocyte gel using the plunger of a 1 mL syringe.
    iii. Once the gels are embedded together, add 1 mL of culture medium to the well and use the tri-culture for further experiments.
      ***Note:*** After 21 days, the adipocyte gel viability starts to decrease to values below 50%–60%, so plan to use the adipocyte gels accordingly.

5. Tetra-culture set up
  The tetra-culture includes four different cell types: HGSOC malignant cell lines, primary fibroblasts, mesothelial and adipocyte cells. In this model, the different cell types are layered on top of the adipocyte gel.
  a. Fibroblasts and mesothelial cell addition
    i. To start layering cells on top of the adipocyte gel, prepare a collagen “adhesive” solution following the recipe outlined in the above “Materials and equipment” section. This is used to stick the adipocyte gel to the bottom of a 96-well plate prior to addition of cells.
    ii. Carefully move the adipocyte gels from 24-well plate to a 96-well plate on a layer (20 μL) of “adhesive” solution.
    iii. Incubate the plate for at least 5 min at 37°C, 5% CO_2_.
    iv. During this time, take an 80%–90% confluent flask of fibroblast, aspirate the spent medium, wash with 1× DPBS followed by trypsin and count the fibroblasts.
    v. Plate the appropriate number of fibroblasts in 100 μL of culture medium on top of the adipocyte gel. (For our model we used 4×10^4^ cells).
    vi. Incubate for 2 h after which wash with 1× DPBS, trypsinize and count the mesothelial cells.
    vii. Plate the appropriate number of mesothelial cells in 100 μL on top of the adipocyte gels with fibroblasts. (For our model we used 2×10^5^ mesothelial cells).
    viii. Incubate for 24 h at 37°C, 5% CO_2_ before proceeding to the next step.
  b. HGSOC cells addition
    i. After 24 h, fibroblasts and mesothelial cells will have attached to the adipocyte gel and formed a nice “mat”.
    ii. Aspirate off the spent medium, wash with 1× DPBS and then de-attach and count the malignant cells.
    iii. Carefully remove the supernatant on the adipocyte gels and replace it with 200 μL of HGSOC cells in culture medium.
    iv. Incubate the gels for 24 h at 37°C, 5% CO_2_.
    v. Gently dislodge the tetra-culture and move to a new 24-well plate with 1 mL of culture medium.

## Expected outcomes

**Figure 2:**
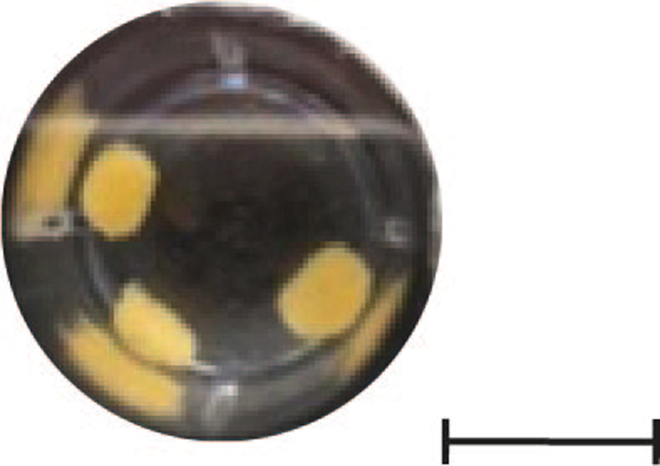
Example of adipocyte gels cultured in a 24-well plate Scale bar: 8 mm.

**Figure 3:**
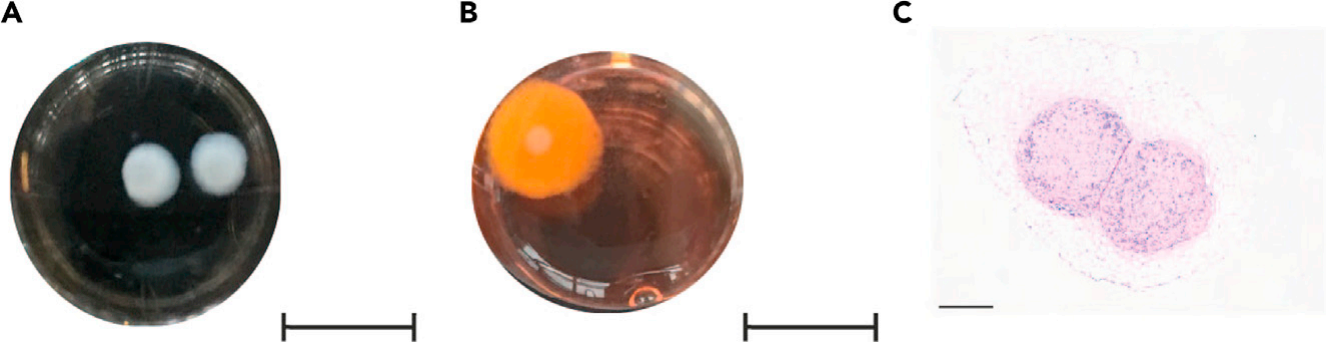
Tri-culture setup (A) Example of collagen gels made up with HGSOC cells and fibroblasts cultured in a 24-well plate. (B) Example of a tri-culture where the collagen gel (white gel in the middle) has been embedded within an adipocyte gel. Scale bar: 8 mm. (C) Tri-culture H&E, scale bar: 500 μm (image adapted from Delaine-Smith et al., 2021).

**Figure 4:**
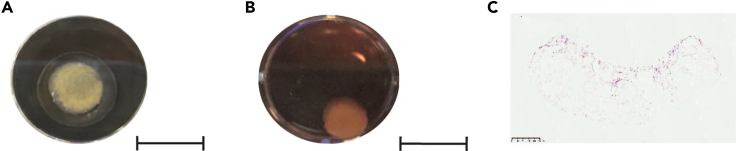
Tetra-culture setup (A) Example of an adipocyte gel anchored to a 96-well plate before the addition of fibroblasts and mesothelial cells. (B) Example of a tetra-culture in 24-well plate 24h after the addition of HGOSC cell. Scale bar: 8 mm. (C) Tetra-culture H&E, scale bar: 500 μm.

A successful tri- or tetra-culture will be viable and usable for experiments up to 21–28 days. An example of adipocyte gel is shown in Figure 2. Figure 3A shows a collagen gel containing fibroblasts and HGSOC cells as detailed in the tri-culture set up protocol 4a. Figure 3B is an example of a complete tri-culture and relative H&E (Figures 3C, (Delaine-Smith et al., 2021)). Figure 4 represents an adipocyte gel (A) and a tetra-culture 24h with relative H&E (B-C) after the protocol completion.

## Limitations

The major limitation of this protocol is the availability of fresh tissue. The quality of the tissue is also very important. In fact, to increase the yield, it is important to process the tissue as soon as possible but within 24 h of surgical de-vascularization.

## Troubleshooting

### Problem 1

The adipocyte yield is not consistent between tissues (1.h).

### Potential solution

The collagenase batch might have changed and needs titration adjustment. To avoid this problem, it is possible to use commercially available Liberase DL, which is composed of a mixture of collagenase enzyme and it is less affected by batch-to-batch variation.

### Problem 2

During the adipocyte isolation, the adipocyte layer is very oily (2.c).

### Potential solution

The quality of the tissue might be not optimal or the minced tissue remained in the collagenase for too long. Try to assess better the quality of the tissue before adding the collagenase solution. If the tissue is soft and fatty, it will require a shorter incubation (20–30 min). If the tissue is stiffer and vascularized, it might require a longer digestion (50 min–1 h).

### Problem 3

The adipocyte gel dissolves after few hours (3.b).

### Potential solution

Add an additional washing step after the filtration to inactivate residual collagenase. Alternatively, the amount of residual medium or oil was too high before casting the gel. Try to reduce at the very minimum both of these conditions by letting the different phases to separate for a longer period.

### Problem 4

Collagen or adipocyte gel mixture solidify before cell addition (3.e).

### Potential solution

The components of the mixture have not been added in the correct order or the rat collagen is too warm or there was a mistake in the addition of the NaOH. Please ensure the order of addition of the components is the same listed in the protocol and check the calculation regarding the volume of NaOH to be added.

### Problem 5

Adipocyte gels will start floating after addition of mesothelial cells or fibroblasts to the model.

### Potential solution

Before adding the next cell type to the model, remove all the media without disturbing the gel and try to make the adipocyte gel adhere to the bottom of the well with a drop of collagen adhesive solution.

## Resource availability

### Lead contact

Further information and requests for resources and data should be directed to the lead contact, Frances Balkwill (f.balkwill@qmul.ac.uk), Barts Cancer Institute, Queen Mary University of London Charterhouse Square EC1M 6BQ, London, UK.

### Materials availability

This study did not generate new unique reagents.

## Data Availability

This study did not generate or analyse any dataset or code.

## References

[bib1] Carswell K.A., Lee M.J., Fried S.K. (2012). Culture of isolated human adipocytes and isolated adipose tissue. Methods Mol. Biol..

[bib2] Delaine-Smith R., Maniati E., Malacrida B., Nichols S., Roozitalab R., Jones R.R., Lecker L.S.M., Pearce O.M.T., Knight M.M., Balkwill F.R. (2021). Modelling TGFβR and Hh Pathway Regulation of Prognostic Matrisome Molecules in Ovarian Cancer. iScience.

[bib3] Malacrida B., Nichols S., Maniati E., Jones R., Delaine-Smith R., Roozitalab R., Tyler E., Thomas M., Boot G., Mackerodt J. (2021). A Human Multi-Cellular Model Shows How Platelets Drive Production of Diseased Extracellular Matrix and Tissue Invasion. iScience.

[bib4] Sonoda E., Aoki S., Uchihashi K., Soejima H., Kanaji S., Izuhara K., Satoh S., Fujitani N., Sugihara H., Toda S. (2008). A new organotypic culture of adipose tissue fragments maintains viable mature adipocytes for a long term, together with development of immature adipocytes and mesenchymal stem cell-like cells. Endocrinology.

[bib5] Tamura N., Shaikh N., Muliaditan D., Soliman T.N., Mcguinness J.R., Maniati E., Moralli D., Durin M.A., Green C.M., Balkwill F.R. (2020). Specific mechanisms of chromosomal instability indicate therapeutic sensitivities in high-grade serous ovarian carcinoma. Cancer Res..

[bib6] Toda S., Uchihashi K., Aoki S., Sonoda E., Yamasaki F., Piao M., Ootani A., Yonemitsu N., Sugihara H. (2009). Adipose tissue-organotypic culture system as a promising model for studying adipose tissue biology and regeneration. Organogenesis.

